# The role of the municipal welfare domain in palliative care: exploring the views of coordinators of Dutch regional palliative care networks

**DOI:** 10.1177/26323524251326188

**Published:** 2025-03-31

**Authors:** Trudy Schutter, Ian Koper, Kris Vissers, Jeroen Hasselaar

**Affiliations:** Department of Primary and Community Care, Radboud UMC, Postbus 9101, 6500 HB Nijmegen, The Netherlands; Agora, Groeneweg 21a, Bunnik 3981 CK, The Netherlands; Department of Primary and Community Care, Radboud University Medical Center, Nijmegen, The Netherlands; Department of Anesthesiology, Pain and Palliative Medicine, Radboud University Medical Center, Nijmegen, The Netherlands; Department of Primary and Community Care, Radboud University Medical Center, Nijmegen, The Netherlands

**Keywords:** focus groups, interdisciplinary collaboration, palliative care, public health palliative care, social support

## Abstract

**Background:**

Collaboration between the healthcare domain and welfare domain could benefit people confronted with an incurable disease residing at home and their informal caregivers, but little is known about this collaboration regarding palliative care. There are regional palliative care networks in the Netherlands, supporting interdisciplinary integrated palliative care; each network has a network coordinator who is a primary liaison for the network and who has an overview of palliative care services and activities in the region. However, the view of the networks on the role of the welfare domain and collaboration with the welfare domain in the field of palliative care is unknown.

**Objective:**

The aim of this study is to explore the awareness of professionals for the social dimension of palliative care and to explore how collaboration between the healthcare domain and the Dutch municipal welfare domain, in the field of palliative care, can be improved.

**Design:**

Focus group research.

**Methods:**

In 2022, six focus groups and two individual interviews were held with 30 coordinators of regional palliative care networks in the Netherlands.

**Results:**

This study showed that coordinators of regional palliative care networks consider collaboration with the welfare domain to be important. There are major differences between the regional palliative care networks regarding knowledge about and collaboration with the welfare domain. Coordinators themselves can function as catalysts for collaboration between palliative care and the welfare domain.

**Conclusion:**

In the Netherlands, collaboration between the welfare domain and the healthcare domain in the field of palliative care is limited and differs considerably between regions. The Dutch municipal welfare domain is relevant for a large group of people confronted with an incurable disease, but it does not provide them with tailored services. Collaboration between palliative care and the municipal welfare domain has great potential, both on the patient level and on the level of the sustainability of palliative care, but it currently seems underexplored.

## Introduction

People who are confronted with an incurable disease, whether as patients, family, friends and/or informal caregivers, may have social needs that require dedicated support beyond the scope of healthcare providers.^1,2^ In the Netherlands, 70% of all the people dying every year die from a non-acute disease trajectory such as cancer, organ failure or dementia^3^; these people could benefit from proactive palliative care since their death is often not unforeseen. Palliative care improves the quality of life of patients and families who are confronted by challenges related to a life-threatening illness, whether physical, psychological, spiritual or social.^4^ Awareness of the social dimension of palliative care requires recognition of the patient’s relationships and the social context in which the disease trajectory and dying are embedded.^2,5–7^ The Dutch quality framework on palliative care acknowledges the importance of the social dimension of palliative care, nevertheless, it does not concretise how addressing social problems and needs should be shaped in practice and by whom.^8^

In the Netherlands, people facing an incurable disease reside at home together with their informal caregivers, as everyone, can request statutory social services from the municipality; this is called the municipal welfare domain. The municipal welfare domain is regulated by different acts, including the Social Support Act.^9,10^ This Social Support Act is aimed at the support of self-reliance of people, making independent living and societal participation possible.^11,12^ Both general services like community centres and shelters and tailor-made services like domestic help, daytime activity programmes, home adjustments, transport facilities and respite care are provided. For the granting of personalised services, the support possibilities of the patients’ own informal network are taken into account.

There are 65 regional palliative care networks in the Netherlands, financed by the Dutch Ministry of Health, Welfare and Sport, to support interdisciplinary integrated palliative care.^13^ These regional networks are formal and sustainable partnerships of independent organisations that are involved with palliative care in a specific geographical region. Each network has a network coordinator who is the primary liaison for communication and coordination between the different formal healthcare providers and informal support organisations. The network coordinator supports the development of the network and improvement of the quality of care within the region of the network and has an overview of palliative care (services) in the region.

The welfare domain and the healthcare domain in the Netherlands are experienced by citizens, professionals and policy makers as separate realms; currently, national policy documents aspire for more collaboration between the two.^14–16^ Collaboration between the two domains could also specifically benefit people confronted with an incurable disease residing at home and their informal caregivers,^17,18^ but little is known about collaboration in the field of palliative care. Therefore, this study explores the awareness of the social dimension of palliative care and how collaboration between the two domains concerning palliative care can be constructed. In line with that, we aim to gain insight into the (potential) role of the welfare domain in the support of people confronted with an incurable disease and to formulate recommendations for collaboration. Since regional palliative care networks are aimed at enhancing interdisciplinary integrated palliative care on a local level, and network coordinators are their primary liaisons, the views and experiences of these coordinators are central in this study.

## Methods

### Study design

This study is part of a larger research project on the role of the Dutch welfare domain in the field of palliative care (SPACE) and a first inquiry of unexplored territory, it could be considered qualitative descriptive as described by Sandelowski.^19,20^ To get a broad insight into the subject, focus groups were held with coordinators of regional palliative care networks in the Netherlands. All network coordinators (*n* = 46; some coordinate multiple networks) were invited by e-mail to participate in an online focus group about the social dimension of palliative care and about collaboration with the municipality and welfare organisations in primary care concerning the support of people confronted with an incurable disease. The focus groups were conducted by TS as a moderator and either IK or JH as an observer. Participant interactions helped expanding the contributions and thus to generate rich data.^21^

The Consolidated Criteria for Reporting Qualitative Research (COREQ)^22^ is used as a guideline to report on this study. In the supplemented checklist, more extensive information about the research team, the study design, and data analysis and reporting can be found (see Supplemental Appendix 1).

### Context and participants

In total, 28 out of 46 coordinators participated in six separate online focus groups. Focus groups had three to six participants and lasted 45–65 min. Two coordinators wished to participate but were not available at the time of the focus group and were, therefore, interviewed individually shortly after (duration was approximately 30 min). Fifteen coordinators declined our invitation due to other appointments, vacations or prolonged illness. One coordinator declined because the topic was not a priority for the regional network. See Table 1 for an overview of participants’ characteristics.

**Table 1.** Participant characteristics.

Characteristics	Participants (*N*)	Participants (%)
Education or work experience		
Nurse, nurse specialist	*N* = 15*	50*
Social worker	*N* = 3*	10*
Other	*N* = 6	20
Unknown	*N* = 7	23
Gender		
Female	*N* = 28	93
Male	*N* = 2	7
Years of experience as network coordinator		
⩽2 years	*N* = 9	30
3–10 years	*N* = 6	20
11–21 years	*N* = 6	20
Unknown	*N* = 9	30

* One participant answered ‘yes’ to both nurse and social worker.

The position of a network coordinator is permanent and part-time; the extent of the employment is based on the population size of a region. Their position is comparable to the position of a project leader, without hierarchal powers, appointed to support the regional palliative care network.^23^

### Data collection

The data collection took place in the spring and autumn of 2022. The focus groups were held digitally via Teams and recorded with images and sound. Participants were invited in writing to participate in the research. This invitation letter provided a general introduction to the topics that were discussed in the focus groups, and the increasing international interest in a public health approach to palliative care that perceives illness, dying, death and care as social events were mentioned as the context of the research. The invitation letter also introduced the Dutch Quality Framework of Palliative Care,^8^ and summarised how this framework presents the social dimension of palliative care. Central in this summary was the fact that patients and their loved ones might experience concerns and problems in relation to their social well-being.

The focus groups consisted of two parts and were guided by a flexible topic list. The first part was about the (awareness of) the social dimension of palliative care in general and was guided by three questions. First, we asked what participants considered to be the social dimension of palliative care, also in relation to the quality framework. Secondly, we asked who or what participants consider to be important for the social well-being of patients and their relatives. And thirdly, we asked to concretise this by asking whose responsibility it is to be aware of the social dimension and to signal social needs. The second part of the focus groups focused on collaboration between the healthcare domain and the welfare domain. We started with open questions on knowledge about the welfare domain and experiences with (collaboration with) the welfare domain. This was followed by questions about collaboration between healthcare professionals and the welfare domain, how this could be stimulated, and whether this is also something the network coordinator has a role in herself. The focus groups were characterised by an open approach to the subject matter and had room for discussion and free response.

### Data analysis

The data from the focus groups was transcribed verbatim. The topic list guided the focus groups and interviews and determined the themes of the findings in that respect; content-wise, the categories and codes were derived from the data by the use of open and axial coding: an inductive thematic analysis was used to obtain important insights grounded in the data itself, without restrains of categories determined beforehand.

Firstly, the data was coded by TS and part of the data was also independently coded by IK. Secondly, the codes were compared and discussed between TS and IK, resulting in a code tree. After that, this was discussed with all authors (TS, IK, KV and JH) in a three hours meeting. As the analysis progressed, codes were refined and relations between codes became increasingly distinct.

### Ethical considerations

Before the start of the study, participants received written information about the study, and informed consent was obtained. Since the study comprised interviews with professionals about their professional’s views and workmanship, the study is not subject to the Medical Research Involving Human Subjects Act (WMO).

As each focus group was organised per consortium (supra-regional organisation of the networks, centred around academic centres of expertise in palliative care), participants often knew each other and sometimes worked together.

## Results

We identified four categories of codes. These are presented as themes in the codebook (see Table 2) and as subtitles in this section.

**Table 2.** Codebook.

Theme	Category	Axial code
1. The social dimension of palliative care	Coordinators awareness about the social dimension of palliative care	Demarcating the social dimension of palliative care from the psychological and existential dimensions is difficult and artificial, but it can be helpful
		The social dimension is about social economic circumstances of people, including finances
		The social dimension is about formal and informal networks and relationships of patients and their relatives
		The social dimension is about who the patient is: his societal life, social identity, daily activities et cetera
	Factors influencing the need for social support	Who the patient is and his/her phase of life influences support needs
		The diagnosis and the phase of the illness trajectory influence support needs
2. The potential role of healthcare providers with regard to the social dimension of palliative care	Assessment of social problems and needs	Healthcare providers should ensure a broad assessment of problems and needs
		Facilitators and barriers to attention of healthcare providers for the social dimension of palliative care
	Referral to welfare organisations	Healthcare providers should assess social problems and needs and listen to patient concerns while being aware that they can refer to others who are more dedicated to these issues
		Healthcare providers should have knowledge of the different social/welfare organisations to be able to refer sensibly
	Collaboration with the welfare domain	Facilitators and barriers to collaboration between the healthcare domain and welfare domain
3. Coordinators’ views of and knowledge about the municipality and the welfare domain	Exploration of and connection with the welfare domain	The welfare domain is organised differently in every municipality, even within one palliative care network, and coordinators are still exploring this domain
		The welfare domain involves a patchwork of various welfare organisations, all sorts of municipal initiatives, respite care, financial support, volunteer organisations et cetera
	Collaboration with municipalities	Municipalities are not familiar with the concept of palliative care but may recognise their role when case studies are presented
		Municipalities and palliative care network coordinators are potential partners
4. Coordinators’ role in stimulating collaboration with the municipality and welfare domain	Sharing knowledge and stimulating awareness about (the social dimension of) palliative care to a greater or lesser extent	Coordinators’ role in stimulating public awareness that life does not stop when an individual is diagnosed with an incurable disease, and that support for people with palliative care needs is not limited to the remit of healthcare professionals
		Coordinators’ role in stimulating awareness about the potential role of the municipality and welfare organisations in palliative care
	Connecting palliative care (providers) with municipalities and welfare organisations to a greater or lesser extent	Coordinators’ role in connecting stakeholders
		Coordinators’ role in stimulating collaboration between the healthcare domain and the welfare domain
		Facilitators and barriers in the partnership between palliative care network coordinators and municipalities

### The social dimension of palliative care

According to the coordinators of palliative care networks, the social dimension of palliative care is about the social and daily life of patients, their social network, relationships, social context, societal position and the social roles they fulfil. Being incurably ill may affect all these activities and roles. For example, being confronted with an incurable disease may affect the possibility to participate in society and the ability to work, affect financial incomes, change the patient’s social position and affect the feeling that he or she matters as a person. The phase of the disease, phase of life of the patient and the preferences of the patient are relevant in this respect, as well as the support possibilities of the social network. Besides, the disease not only affects the individual patient but also his or her relatives. Some coordinators link the social dimension of palliative care to societal views on caregiving and dying also, for example by discussing the willingness of people to provide care for each other.

On a meta level, two comments were frequently made about the social dimension of palliative care. Coordinators typically adhere to the division of palliative care into four dimensions, namely the physical, psychological, spiritual and social dimensions; however, they consider this division artificial and flexible and perceive that the social dimension is intertwined with all other dimensions rather than a separate thing. Further, when asked about the social dimension of palliative care, coordinators refer regularly to the concept of ‘positive health’,^24,25^ a model with a broad view of health, mentioning that municipalities and welfare organisations are familiar with this concept as well.

### The potential role of healthcare providers with regard to the social dimension of palliative care

According to the network coordinators, healthcare providers in primary care, such as the GP and the community nurse, need to be aware that their patients may have or develop social needs, and should explore those and refer accordingly. Although coordinators support this signalling and referring role for healthcare providers in relation to social needs, they realise that this is not always a reality or possibility due to the workload and labour shortage within healthcare. Yet, one coordinator emphasised that this labour issue necessitates collaboration with professionals and organisations other than healthcare providers. To refer patients appropriately, healthcare providers need to know which social organisations and initiatives exist and what they offer. Another coordinator remarked that patients potentially contact welfare organisations or the municipality themselves, but more emphasis was placed on contact via healthcare providers since these are often contacted first.

There is considerable agreement between coordinators that healthcare professionals do not have to provide extensive social support themselves. Nevertheless, they should be aware of the ‘whole picture’: that the patient is more than his or her medical illness or condition. The communication style of healthcare professionals seems to be pivotal to open discussion and recognition of patients’ social problems. Patients can be ashamed to mention certain problems, especially financial ones; coordinators expect that a reliable long-term relationship between the healthcare provider and the patient is helpful in such cases. Further, attention to the physical environment and housing of patients can give clues about their social situation and thus be helpful in recognising needs.

Factors brought up that could stimulate attention to the social dimension are palliative care case managers and the use of advanced care planning conversations. Factors reported that complicate attention to social aspects include the fact that patients can move through the whole healthcare system, the absence of one clear responsible healthcare professional for social concerns and the high workload of healthcare professionals.

### Coordinators’ views of and knowledge about the municipality and the welfare domain

All participants agreed on the importance of a position for welfare organisations in the support of people with palliative care needs. Support by professionals from the welfare domain can potentially relieve the burden on the healthcare domain, including palliative care providers. One coordinator noticed welfare professionals’ potential role in signalling healthcare needs, since welfare professionals are sometimes earlier involved and may, therefore, already have built a relationship. Many coordinators note not being sure about all the services and possibilities of the welfare domain and how to gain access to this domain. Between coordinators, there are major differences in the amount of knowledge about and collaboration with the welfare domain. Some mention not having any contact with welfare organisations or municipalities; others refer to it as an important topic in their annual plan and have affiliated welfare organisations in their palliative care network. A number of coordinators indicated that national guidance on these topics would be helpful.

When asked about who or what the welfare domain consists of, coordinators brought up the Social Support Act and other laws that are implemented by the municipality, the arrangements covered by these laws, and the organisations and professionals that execute these. Further, coordinators discussed volunteers, funeral organisations, centres for existential questions, and financial and legal support.

### Coordinators’ role in stimulating collaboration with the municipality and welfare domain

Most network coordinators see it as their role to draw attention to the social dimension of palliative care and to facilitate the connection between palliative care providers and municipalities and welfare organisations. Many coordinators emphasise that life does not stop when someone is diagnosed with an incurable disease and affirm that the social life and social network of a patient are relevant and affect the quality of life of a patient. In this role, some coordinators focus mostly or only on ‘the work floor’ and thus on cooperation at the operational level, while others have elaborate contacts at the organisational level of welfare organisations and at the policy level with municipalities.

Coordinators that aim to connect palliative care with municipal policy accomplish this by contacting the alderman, by joining consultation meetings or policy sessions, by sharing knowledge about palliative care and by pointing municipalities their responsibilities and possibilities to collaborate. These coordinators notice that it is often unclear for the municipality whether and how they have a responsibility for people with palliative care needs; a coordinator reported that presenting a case study turns out to be helpful then. Further, understanding each other’s world appears to be effective: it is helpful when the coordinator comes prepared, with a clear message and idea of how to translate this into municipal policy; and when the alderman or policymaker has personal experience or affinity with the topic, this seems to soothe potential cooperation. On the benefit side, some coordinators experienced that collaboration with the municipality can be helpful in housing a hospice and supportive in the organisation of activities focused on societal awareness around death and dying, for example by subsidising and promoting death cafes.

Coordinating a palliative care network is often not a full-time position, some coordinators combine the coordination of several palliative care networks and others are also coordinators of a centre for existential questions or of a dementia network; these other activities seem to positively impact knowledge about the welfare domain and access to the municipality. Coordinators have the impression that other existing networks, such as the dementia network and the municipality, are more used to exchange and to collaborate than palliative care networks. They point out that municipalities are responsible for day activity programmes for people with dementia living at home and are often familiar with the concept of dementia-friendly communities; some coordinators even suppose a form of competition between municipalities in becoming the most dementia friendly and report that dementia is more experienced as a societal problem.

### Participant quotations

In Table 3, participant quotations are presented to illustrate the findings.

**Table 3.** Illustrative quotes, categorised per theme of the codebook.

**Theme 1: The social dimension of palliative care**	
Q1.1	‘*I have interpreted the social dimension as the societal life of someone and everything attached to that, which can be as narrow or broad as someone’s life or the phase of life they are in. So it is different for a child than for a frail elderly person, their social part will be shaped differently as well*’. (NC 6 in FG 2)
Q1.2	‘*It’s not about being ill, it is about seeing a human being. Moving from care and support to welfare and happiness. We are so focused on care and support, but it is the welfare part that has a huge impact*’. (NC 24 in FG 5)
**Theme 2: The potential role of healthcare providers with regard to the social dimension of palliative care**	
Q2.1	‘*The GP should be able to do that [investigate beyond physical symptoms], it is a generalist, she needs to notice these things, know social organisations and refer appropriately. In inpatient clinics, this is much less likely. In a hospital, you come for a treatment and then you leave, and you would hope a nurse would notice something. And that does happen. But eh, the GP is at the core*’. (NC 1 in FG 1)
Q2.2	‘*[In our region] we work with palliative care case managers. Most of the support they provide, if I had to put it in those dimensions, would be in the social part. They refer people to the welfare domain very often, but they also support them with practical questions like what to expect, or related to their loved ones. So I see that this really resonates with the patients, and that support in this respect is very much needed*’. (NC 8 in FG 2)
Q2.3	‘*Actually, I feel that – thinking out loud here – it [social aspects] belongs to everyone [all health care professionals]. In the same way awareness of existential questions belongs to every generalist. And the moment you struggle with that, there are welfare organisations who specialise in these things. Or if you are an informal caregiver, there are organisations to support you. You can get specific support or expertise there. I think it works like that, just like those centres for existential questions*’. (NC 2 in FG 1)
**Theme 3: Coordinators’ views of and knowledge about the municipality and the welfare domain**	
Q3.1	‘*I am also coordinator of the centre for existential questions in this region, and in that respect I get in touch with the welfare domain quite often. And since I took that role, I noticed how broad it is, where previously I didn’t know. Now I have frequent contacts with community teams, social teams, all kinds of municipal initiatives, to improve public health from the perspective of the welfare domain. That’s a world I didn’t know coming from the palliative care network*’. (NC 10 in FG 2)
Q3.2	‘*It is all so fragmented, that is also a very big problem. [All professionals] take very good care of their own shop and [. . .] have their hands full with that too. But, we are no longer inclined to help each other. I think that that’s a very important role for us a network coordinators, and that we know all those parties. That is also why there is the desire [from the palliative care network] to get to know the welfare domain better and to involve it too, because we have the opportunity to bring those parties together*’. (NC 16 in FG 4)
**Theme 4: Coordinators’ role in stimulating collaboration with the municipality and welfare domain**	
Q4.1	‘*The connections I have with the municipalities from the palliative care network are quite limited. However, because I am also involved in dementia care, we actually work together a lot with the municipalities. And that of course also has to do with the fact that municipalities have an important task within dementia care, for example with day care, because they provide those indications for the Social Support Act. [. . .] So I have to say, cooperation is close, also around existential care. So that part, yes. But now and then I let something slip about palliative care and then they say*: “*yes, that. . .what should we as a municipality do about that?*”’. (NC 6 in FG 2)
Q4.2	‘*[my role is:] Making those connections. Getting them together. It can be a local meeting or a regional meeting, but getting those stakeholders together is our role, and it is very difficult. I have two municipalities joined in our steering group [of the palliative care network], but by the time I knew who to turn to I had been sent from pillar to post, because nobody thinks palliative care is their business [. . .] You just have to find someone [from the municipality] who’s interested in the topic*’. (NC 23 in FG 5)
Q4.3	‘*I hardly cooperate with the welfare domain. Sometimes I try something with a welfare organisations, but it’s difficult. I’ve been invited to tell something about palliative care at a welfare organisation, and I’ve been called by a welfare providers, for a patient who wanted to stop eating and drinking and she wanted to know something about that*’. (NC13 in FG3)

## Discussion

In this study, we explored awareness of the social dimension of palliative care, and the role of the Dutch municipal welfare domain in the support of people confronted with an incurable disease living at home, disclosed by coordinators of Dutch regional palliative care networks. We also explored the connection between palliative care and the welfare domain. This study shows that network coordinators consider the social dimension of palliative care and collaboration with the welfare domain to be important items; that there are major differences between the regional palliative care networks regarding knowledge about and collaboration with the welfare domain; and that coordinators themselves can function as catalysts for collaboration between palliative care and the welfare domain.

### Strengths and limitations

A major strength of this study lies in its design. In the online focus groups, we were able to include network coordinators from all palliative care networks in the Netherlands, gathering a nationwide perspective on the role of the welfare domain in palliative care. Still, it is possible that mainly network coordinators with a special interest in the social dimension of palliative care participated, leaving the perspective of those with no interest in the welfare domain underrepresented. Additionally, while online focus groups are more practical and easily organised and accessed, the interaction between participants is thought to be slightly more limited than in traditional focus groups, although the difference is being eroded.^26^ Since each focus group was organised per consortium, participants often knew each other already and sometimes worked together. We consider this to have been a positive influencing factor that improved openness in the discussion; some participants expressed that the mutual exchange was inspiring for their own practice and future actions with regard to the municipal welfare domain.

### The role of the municipal welfare domain in palliative care

In the Netherlands, the services and facilities provided under the Social Support Act are relevant for a large group of people confronted with an incurable disease, but the municipal welfare domain is broader than these specific services. The primary target group within the municipal welfare domain is vulnerable citizens, and welfare professionals seem to function as their support network or their liaison to support sources. Theoretically, welfare professionals may function as the voice of citizens who are confronted with an incurable disease, consider their social context and influencing environmental factors, and reach out to family and significant others.^17,27^ In practice, however, this is not self-evident.^28^ In supporting patients with palliative care needs specifically, it is helpful when welfare professionals have basic knowledge about common palliative care trajectories and the availability of specific (volunteer) services aimed at support at the end of life.^28^ Hospices in example the United Kingdom and Ireland play a central role in the provision of palliative social support in the community, working alongside the healthcare and social care system.^29^ Unlike these, the Dutch municipal welfare domain does not provide specific services tailored to people confronted with an incurable disease, and there is no familiarity with for example palliative day care or palliative social work. Yet, outside the remit of the municipal welfare domain, local initiatives aimed at palliative social support can be found, provided for example volunteer organisations, churches and disease-specific foundations.

### The role of network coordinators in palliative care collaboration

According to the network coordinators, healthcare providers should be able to recognise social support needs in their patients with incurable diseases, and refer them to social support service providers accordingly. This promotes addressing the social dimension of palliative care. However, earlier research showed that over half of the Dutch GPs do not involve social welfare services when providing palliative care.^30^ This emphasises the opportunity for network coordinators as a liaison between the healthcare and welfare domains, making healthcare providers aware of the availability and the added value of the services and facilities within the welfare domain. This requires that network coordinators have knowledge about the position and role of the (municipal) welfare domain in general and learn from experiences with the welfare domain on the topic of palliative care specifically, for example by mutually exchanging these experiences with other coordinators in their consortium. With extensive knowledge of the available services and facilities within their network, the coordinator, or where available the palliative care case manager, could then serve as a navigator for healthcare providers looking for social support for their patients with incurable disease, much like the Nav-CARE volunteers do for patients and families.^31^ However, as some network coordinators acknowledge that there is little cooperation with the welfare domain (see also quote Q4.3), there seems still much to gain in this area. Often, the welfare domain is considered by coordinators to be a new territory, and its potential in supporting patients nearing the end of life and their families has not yet been unlocked.

Meaningful collaboration between healthcare providers and the welfare domain requires a clear vision of the added value of the welfare domain in palliative care.^32^ In the argument that caregiving, dying, death and bereavement are social events with medical aspects rather than medical problems with social aspects,^33,34^ a societal point of view on palliative care could help normalise death and increase community engagement in the support of people confronted with an incurable disease. Also, this approach could strengthen the vision of the added value of collaborating with the welfare domain on the topic of palliative care.

### Further research

Further research should be focused on the viewpoint and practical actions of municipalities and welfare organisations on subjects like caregiving, dying, death and bereavement. Both the individual level as well as the level of public awareness deserve appropriate research attention. With increased awareness of the social dimension of palliative care, more people who are confronted with an incurable disease and who also have social needs may find their way to the municipal welfare domain. This may strengthen appropriate social support and improve the sustainability of palliative care for those in need.^35^

## Conclusion

Coordinators of regional palliative care networks consider the social dimension of palliative care and collaboration with the welfare domain to be important items. Currently, in the support for people confronted with an incurable disease, the healthcare domain and the municipal welfare domain only collaborate to a limited extent. To broaden this, future collaborations may open up new and extended possibilities for the support of people confronted with incurable diseases. Coordinators of regional palliative care networks can catalyse local collaboration and connect local developments at an aggregate level. In this way, they help concretising attention to the social dimension of palliative care as presented by the Dutch quality framework on palliative care.

## Supplemental Material

sj-docx-1-pcr-10.1177_26323524251326188 – Supplemental material for The role of the municipal welfare domain in palliative care: exploring the views of coordinators of Dutch regional palliative care networks Supplemental material, sj-docx-1-pcr-10.1177_26323524251326188 for The role of the municipal welfare domain in palliative care: exploring the views of coordinators of Dutch regional palliative care networks by Trudy Schutter, Ian Koper, Kris Vissers and Jeroen Hasselaar in Palliative Care and Social Practice

## Declarations

**Ethics approval and consent to participate:** Ethics approval is waived off by the Medical Ethical Committee East Netherlands (file number 2021-13359) since the study is not subject to the Medical Research Involving Human Subjects Act. The study comprised interviews with professionals about their professional’s views and workmanship. Written consent for participation was gained prior to the focus groups and interviews commencing.

**Consent for publication:** Not applicable.

**Availability of data and materials:** The data generated and/or analysed during the current study are available from the corresponding author on reasonable request.
