# Supplementary material for: The role of the municipal welfare domain in palliative care: exploring the views of coordinators of Dutch regional palliative care networks
Source: Palliat Care Soc Pract. 2025 Mar 31;19:26323524251326188. doi: 10.1177/26323524251326188 (PMC11956517; doi:10.1177/26323524251326188)
Supplement: sj-docx-1-pcr-10.1177_26323524251326188 – Supplemental material for The role of the municipal welfare domain in palliative care: exploring the views of coordinators of Dutch regional palliative care networks [file sj-docx-1-pcr-10.1177_26323524251326188.docx]

| No | Item | Guide questions/description |
| --- | --- | --- |
| Domain 1: Research team and reflexivity | | |
| Personal Characteristics | | |
| 1. | Interviewer/facilitator | The focus groups were conducted by TS, together with either IK or JH. The two individual interviews were conducted by TS. |
| 2. | Credentials | TS – MA MSc  IK – PhD, senior researcher in palliative care  KV – PhD, MD, professor pain and palliative medicine  JH – PhD, professor of social empowerment in palliative care |
| 3. | Occupation | All researchers had an occupation at Radboudumc at the time of the study |
| 4. | Gender | TS – female  IK, KV and JH - male |
| 5. | Experience and training | TS is educated in humanities and health care sciences, and experienced as spiritual caregiver and moderator of moral case deliberations. She received training on Qualitative research methods and analysis.  IK, KV and JH are all senior researchers. IK is educated in health care sciences, KV in medicine and JH in health care sciences and ethics. |
| Relationship with participants | | |
| 6. | Relationship established | Prior to the study, there was a familiarity between some of the researchers and some of the participants, as a result of the relatively small palliative care field in the Netherlands. |
| 7. | Participant knowledge of the interviewer | At the start of the focus groups and interviews, there was a small introduction moment. Participants were told some of the background of the conducting researchers and the reasons for doing the research. |
| 8. | Interviewer characteristics | At the start of the focus groups and interviews, it was reported that the researchers had the assumption that there could be a role for the municipal welfare domain in the support of people with palliative care needs, especially on the social dimension of palliative care. |
| Domain 2: study design | | |
| Theoretical framework | | |
| 9. | Methodological orientation and Theory | The study was a qualitative descriptive research in the orientation of Sandelowski (Sandelowski, 2000; 2010) |
| Participant selection | | |
| 10. | Sampling | There was no selection, all network coordinators of Dutch regional palliative care networks (n=46) were approached to participate in this research. |
| 11. | Method of approach | Participants were approached by email |
| 12. | Sample size | There were 30 participants in the study (65%) |
| 13. | Non-participation | 15 potential participants declined our invitation due to other appointments, vacation, or prolonged illness; 1 potential participant declined because the topic was not a priority for the regional network.  There were no participants that dropped out. |
| Setting | | |
| 14. | Setting of data collection | The data was collected digitally since the focus groups were held online via Teams meetings. |
| 15. | Presence of non-participants | There was no one present beside the participants and researchers |
| 16. | Description of sample | The sample consisted solely of network coordinators of Dutch regional palliative care networks |
| Data collection | | |
| 17. | Interview guide | The focus groups and interviews were guided by a topic list consisting of mainly open questions. As the data collection progressed, additional questions were added to the topic list, to discuss findings of earlier focus groups. |
| 18. | Repeat interviews | There were no repeat interviews carried out. |
| 19. | Audio/visual recording | The data was collected digitally via Teams by the use of audio and visual recording |
| 20. | Field notes | Small field notes were made during and after the focus groups |
| 21. | Duration | Focus groups lasted 45 to 65 minutes, the individual interviews were approximately 30 minutes. |
| 22. | Data saturation | With 65% of all Network Coordinators participating in our study, we could of have missed specific examples, but we consider data to be saturated, especially since the study had an explorative aim. |
| 23. | Transcripts returned | Transcripts were not returned to participants for comment and/or correction |
| Domain 3: analysis and findings | | |
| Data analysis | | |
| 24. | Number of data coders | All authors were involved with the data analysis. The data was coded by two data coders: TS coded all data, and part of the data was also independently coded by IK. |
| 25. | Description of the coding tree | The coding tree is described in the code book, provided in table 2 of the manuscript. |
| 26. | Derivation of themes | The topic list guided the focus groups and interviews and determinated the themes of the findings in that respect. Content-wise, the categories and codes were derived from the data. |
| 27. | Software | Atlas.ti 24.0.0 was used to manage the data |
| 28. | Participant checking | One participant was approached for and provided feedback on the findings |
| Reporting | | |
| 29. | Quotations presented | Participant quotations were presented to illustrate the themes / findings. Each quotation was identified. |
